# Comparative effects of dietary functional nutrients on growth performance, meat quality, immune responses, and stress biomarkers in broiler chickens raised under heat stress conditions

**DOI:** 10.5713/ab.21.0230

**Published:** 2021-08-21

**Authors:** Deok Yun Kim, Jong Hyuk Kim, Won Jun Choi, Gi Ppeum Han, Dong Yong Kil

**Affiliations:** 1 Department of Animal Science and Technology, Chung-Ang University, Anseong 17546, Korea

**Keywords:** Broiler Chicken, Functional Nutrient, Growth Performance, Heat Stress, Immune Response, Stress Biomarker

## Abstract

**Objective:**

The objective of the present study was to investigate the comparative effects of dietary functional nutrients including glutamine (Gln), chromium picolinate (Cr picolinate), vitamin C (Vit C), betaine (Bet), and taurine (Tau) on growth performance, meat quality, immune responses, and stress biomarkers in broiler chickens raised under heat stress conditions.

**Methods:**

A total of 420 21-d-old Ross 308 male broiler chickens (initial body weight = 866±61.9 g) were randomly allotted to 1 of 7 treatment groups with 6 replicates. One group was kept under thermoneutral conditions and was fed a basal diet (PC, positive control). Other 6 groups were exposed to a cyclic heat stress condition. One of the 6 groups was fed the basal diet (NC, negative control), whereas 5 other groups were fed the basal diet supplemented with 0.5% Gln, 500 ppb Cr picolinate, 250 mg/kg Vit C, 0.2% Bet, or 1.0% Tau. The diets and water were provided *ad libitum* for 21 d.

**Results:**

Broiler chickens in NC group had decreased (p<0.05) growth performance and immune responses measured based on cutaneous basophil hypersensitivity (CBH), but increased (p<0.05) stress responses measured based on feather corticosterone concentrations and blood heterophil:lymphocyte than those in PC group. However, none of dietary functional nutrients had a positive effect on growth performance of broiler chickens. Dietary supplementation of 250 mg/kg Vit C improved (p<0.05) CBH responses of broiler chickens, but other functional nutrients had no such an improvement in CBH responses. All functional nutrients decreased (p<0.05) stress responses of broiler chickens.

**Conclusion:**

Functional nutrients including Gln, Cr picolinate, Vit C, Bet, and Tau at the supplemental levels used in this study decrease stress responses of broiler chickens to a relatively similar extent. However, this reduction in stress responses could not fully ameliorate decreased productive performance of broiler chickens raised under the current heat stress conditions.

## INTRODUCTION

Heat stress is one of the major problems in the current poultry industry because of steadily increasing environmental temperature worldwide. Heat stress results from increasing heat loads in the body by increasing heat production with limited heat loss. Poultry have the least capacity of dissipating heat because of a small number of sweat glands and feather covering, which makes poultry the most sensitive to heat stress among livestock animals [1]. In addition, recent intensive breeding strategies realize the very high performance of poultry, whereas it concomitantly causes increased heat production, which lowers the ability of thermoregulation of poultry [2]. Poultry exposed to heat stress is well-known to experience various physiological and behavioral abnormalities, which markedly decrease productive performance, health, and welfare of poultry [3–6]. Thus, the development of effective strategies to decrease the heat stress of poultry is essential for the current and future poultry industry.

Dietary managements with modified energy and nutrient concentrations in diets can be one potential solution for heat stress-associated problems in poultry. The practical and convenient approach may be an increasing supplementation of functional nutrients that play a specific role in mitigating physiological and metabolic disorders as caused by heat stress [5]. Among various functional nutrients, dietary supplementation of glutamine (Gln), chromium (Cr), vitamin C (Vit C), betaine (Bet), and taurine (Tau) has gained attention because of their positive effects on productive performance and health of broiler chickens raised under heat stress conditions [5–9]. Feeding diets supplemented with 0.5% to 1.0% Gln [7,8,10,11], 500 to 2,000 ppb Cr [12], 250 to 500 mg/kg Vit C [13–15], 0.05% to 0.2% Bet [16], or 0.5% to 1.0% Tau [17,18] was reported to ameliorate growth depression and health problems of broiler chickens raised under heat stress conditions, possibly by improving immune responses, antioxidant activity, anti-inflammatory responses, and nutrient utilization; however, the results remained inconclusive [19]. Furthermore, to our knowledge, no studies have compared the effects of these functional nutrients on the anti-stress responses of broiler chickens.

Therefore, the objective of the present study was to com pare the efficacy of dietary functional nutrients including Gln, Cr, Vit C, Bet, and Tau on growth performance, meat quality, immune responses, and stress biomarkers in broiler chickens raised under heat stress conditions.

## MATERIALS AND METHODS

### Animals, experimental design, and diets

The protocol for the current experiment was reviewed and approved by the Institutional Animal Care and Use Committee at Chung-Ang University (IACUC No. 2020-00022).

A total of 600 Ross 308 male broiler chicks (1 d of age) were obtained from a local hatchery (Dongsan broiler hatchery, Cheonan-si, Korea). All chicks were fed a commercial diet containing adequate energy and nutrients [20] before the start of the experiment. On 21 d of age, all broiler chickens were weighed and 180 broiler chickens with extremely high and low body weight (BW) were discarded. The remaining 420 broiler chickens with an average BW of 866± 61.9 g were allotted to 1 of 7 treatment groups with 6 replicated cages in a completely randomized design. Each cage had 10 broiler chickens. The chickens in one group were raised under thermoneutral conditions at 20°C (degree Celsius) and 57% relative humidity during all experimental period and were fed a basal diet (PC, positive control). The chickens in other 6 groups were exposed to a cyclic heat stress condition at 31°C to 32°C for 8 h/d and 27°C to 28°C for the remaining time. The chickens in 1 of 6 groups under the heat stress conditions were fed the basal diet as a negative control (NC), whereas those in 5 other groups were fed the basal diet supplemented with 0.5% Gln (99%, Neo-Cremar, Seoul, Korea), 500 ppb Cr picolinate as a Cr source (3,000 ppm, Easybio, Seoul, Korea), 250 mg/kg Vit C (97%, Genebiotech, Seoul, Korea), 0.2% Bet (98%, Genebiotech, Korea), or 1.0% Tau (98%, Genebiotech, Korea). The basal diet was formulated to meet or exceed the nutrient recommendations of the Ross 308 manual [20] and various functional nutrients including Gln, Cr, Vit C, Bet, and Tau were added to the basal diet at the expense of celite. The supplemental levels of the functional nutrients in the present study were determined based on the results of the previous studies reporting their positive effects on heat stress responses of poultry.

All diets were fed to broiler chickens on an *ad libitum* basis for 21 d. A 23-h lighting program was used during the experiment. The BW, body weight gain (BWG), and feed intake (FI) were recorded at the end of the experiment. The mortality was recorded daily, and the feed conversion ratio (FCR) was calculated as FI divided by BWG after the correction for mortality [21].

### Sample collection and analysis

At the end of experiment (42 d of age), the individual weights of all chickens were recorded. Five birds with a BW close to the average BW in each replicate cage were selected. One bird was euthanized by CO_2_ asphyxiation to analyze breast meat quality. Two birds were used to analyze immune responses, whereas the other 2 birds were used to analyze stress biomarkers.

For meat quality, the right portion of the breast meat was excised to measure the pH [18], meat color including lightness (L*), redness (a*), and yellowness (b*), water holding capacity (WHC) at 24 h postmortem [22], and the thiobarbituric acid-reactive substances (TBARS) at 7 d after storage at 4°C. The detailed procedure of meat quality analysis was reported previously [21,23].

Cutaneous basophil hypersensitivity (CBH), a measure of the cell-mediated immune responses, was determined based on the method of Corrier and DeLoach [24]. In short, the analysis was conducted by intradermally injecting 100 μg phytohemagglutinin-P (PHA-P) in 0.10 mL of saline solution into each bird between the third and fourth digits of the right foot. The left foot of each bird was also injected with 0.10 mL of phosphate-buffered saline (PBS) (pH 7.4) as the control. Toe skin thickness was measured using a micrometer (Fisher Scientific Co., Pittsburgh, PA, USA) at 6 and 12 h post-injection with PHA-P. The values for CBH responses were calculated as follows [24]:

CBH response=(RFA-RFB)-(LFA-LFB)

where, CBH response = skin thickness (mm), RFA = right foot skin thickness after injection, RFB = right foot skin thickness before injection, LFA = left foot skin thickness after injection, and LFB = left foot skin thickness before injection.

Blood heterophil:lymphocyte (H:L) was measured as a stress biomarker. The analysis of blood H:L was performed based on the method reported previously [25,26]. In short, blood samples were collected from the wing veins of the selected birds. Approximately 7 μL of blood were smeared on a standard microscope slide. The slides were dried at a room temperature and post-fixed with methanol for 5 min. The smear was stained in 0.2 mL of Wright stain (Muto Pure Chemicals, Tokyo, Japan; Wright stain solution) for 2 min at a room temperature, and then was rinsed with water. The smear was stained again using 0.2 mL of Giemsa stain (Duksan Pure Chemicals, Ansan, Korea; Giemsa’s staining solution) for 5 min at a room temperature and then was rinsed with water. Following air drying, the hemocyte smear was inspected under a microscope. Blood H and L numbers were counted by the same person (up to 100 cells per individual smear) and the blood H:L was calculated.

The primary flight feather samples were also collected from the same birds used to analyze blood H:L. Feather corticosterone (CORT) concentrations as a measure of accumulative stress hormones in the chicken were analyzed [27,28]. The analysis of feather CORT concentrations was performed based on the method reported previously [29] with minor modification. Briefly, the calamus was removed and the feather vanes minced into pieces of less than 5 mm^2^ with scissors. A total of 10 mL of methanol (HPLC grade, Honeywell, NC, USA) was added and the samples were placed in a sonication water bath at a room temperature for 30 min, followed by incubation at 50°C overnight in a shaking water bath. The methanol was then separated from feather materials using a syringe filter (HyunDai Micro, Anseong, Korea) in a filtration funnel. The feather remnants were washed twice with 2 mL of methanol; the washes were added to the original methanol extract. The methanol extract was placed in a water bath at 50°C and subsequently evaporated in a fume hood under air circulation. Evaporation of the samples was completed within a few hours and the extract residues were mixed with a 1 mL of the PBS (pH 7.4). The CORT concentrations in the mixed sample were measured using a corticosterone competitive ELISA kit according to the manufacturer’s protocol (Thermo Fisher Scientific, USA). The feather CORT concentrations were expressed as a function of the feather length (pg/mm) [28].[Table t1-ab-21-0230]

### Statistical analysis

All data were analyzed by analysis of variance as a completely randomized design using the PROC MIXED procedure (SAS Institute Inc., Cary, NC, USA). The replicate was considered as an experimental unit for all measurements. Outlier data were checked using the PROC UNIVARIATE procedure of SAS; however, no outlier was identified. The LSMEANS procedure was used to calculate treatment means and the PDIFF option of SAS was used to separate the means if the difference was significant. Statistical significance was considered at p<0.05.

## RESULTS

### Growth performance

Broiler chickens in all 6 heat-stressed groups had decreased (p<0.05) growth performance including final BW, BWG, and FI than those in PC group (Table 2). Among heat-stressed groups, broiler chickens fed diets supplemented with 0.5% Gln had greater (p<0.05) final BW and BWG than those fed diets supplemented with 500 ppb Cr picolinate; however, other treatments (i.e., 250 mg/kg Vit C, 0.2% Bet, and 1.0% Tau) showed no differences in final BW and BWG as compared to Gln and Cr picolinate treatments. Neither environmental conditions nor dietary functional nutrients influenced the FCR of broiler chickens.

### Meat quality

Breast meat of broiler chickens in NC group showed less (p<0.05) pH at 24 h postmortem than in PC group (Table 3). Under heat stress conditions, feeding diets supplemented with 0.5% Gln, 500 ppb Cr picolinate, 0.2% Bet, or 1.0% Tau increased (p<0.05) pH of breast meat at 24 h postmortem than feeding the basal diet, but such an effect was not significant for feeding diets supplemented with 250 mg/kg Vit C. However, other measurements including WHC, meat color (i.e., L*, a*, and b*), and TBARS values were not affected by environmental conditions and dietary functional nutrients.

### Immune responses

The CBH responses measured at 6 h post-injection with PHA-P did not clearly differ among treatment groups (Table 4). Broiler chickens in Vit C treatment had the greatest (p< 0.05) CBH responses but those in 500 ppb Cr picolinate treatment had the least (p<0.05) CBH responses at 6 h post-injection with PHA-P. However, at 12 h post-injection with PHA-P, broiler chickens in NC group had less (p<0.05) CBH responses than those in PC group. Under heat stress conditions, feeding diets supplemented with 250 mg/kg Vit C had a greatest (p<0.05) CBH responses than feeding the basal diet or diets supplemented with other functional nutrients, with this effect being similar to that observed in PC group. However, feeding diets supplemented with 0.5% Gln, 500 ppb Cr picolinate, 0.2% Bet, or 1.0% Tau had no positive effects on CBH responses under heat stress conditions.

### Stress biomarkers

Broiler chickens in NC group had greater (p<0.05) feather CORT concentrations and blood H:L than those in PC group (Table 5). Under heat stress conditions, dietary supplementation of all functional nutrients decreased (p<0.05) feather CORT concentrations and blood H:L although the effect was not significant for 1.0% Tau treatment with regard to feather CORT concentrations and for 250 mg/kg Vit C treatment with regard to blood H:L. Among 5 functional nutrients, feeding diets supplemented with 0.2% Bet and 1.0% Tau showed the least feather CORT concentrations and blood H:L, respectively, which were comparable to those observed in PC group.

## DISCUSSION

The heat stress has been reported to impair growth performance of poultry, leading to a significant economic loss in poultry production [30–33]. Similar results for decreased growth performance were observed in the current study. The reduction in growth performance of poultry exposed to heat stress is largely attributed to the loss of appetite [34], decreased digestion, absorption, and utilization of dietary nutrient and energy [35,36], endocrine disorders, systemic immune dysregulation, abnormal behavior, and increased oxidative stress [3,37,38]. Many efforts have been made to minimize this negative impact on poultry performance by dietary supplementation of various functional nutrients.

Dietary supplementation of 0.5% to 1.0% Gln has been reported to improve growth performance of broiler chickens exposed to heat stress [7,8,10,11]. The possible mechanisms underlying this positive effect of Gln are primarily associated with improved intestinal function and health by increased intestinal cell development and enzyme activity and modulated immune responses and microbial populations in animals [39,40].

Dietary Cr is an essential mineral related to the proper insulin function, which directly affects various nutrient metabolism in animals [41,42]. Under heat stress conditions, beneficial effects of dietary supplementation of 500 to 2,000 ppb Cr on anti-stress responses for broiler chickens have been reported, which is mainly linked to improved nutrient metabolism, antioxidant activity, and immune responses [12].

Dietary Vit C is a potent biological antioxidant and many previous experiments have demonstrated that dietary supplementation of Vit C is effective in decreasing the oxidative stress, and therefore, has a positive effect on poultry performance, especially when poultry are exposed to high oxidative stress such as heat stress [9,13–15]. It was reported that dietary supplementation of 250 mg/kg Vit C may be optimal to improve broiler performance under heat stress conditions [14].

Dietary Bet can act as an active osmolyte to maintain water and ion balance, thereby improving the capacity of poultry to adjust to dehydration and osmotic imbalance resulting from heat stress [16,42]. In addition, Bet has a methyl donor property, which is related to enhanced intestinal development and nutrient utilization in the animal body [16,43]. Therefore, dietary supplementation of 0.05% to 0.2% Bet has shown beneficial effects on growth performance of broiler chickens exposed to heat stress [16].

Dietary Tau is a sulfur-containing amino acid with vari ous biological functions, such as membrane stabilization, bile acid conjugation, vitagene activation, anti-inflammation, and immune stimulation in animals [18,19]. Dietary supplementation of 0.5% to 1.0% Tau has been reported to restore heat stress-induced growth impairments and physiological dysfunctions in broiler chickens [17,18,44].

Although dietary supplementation of Gln, Cr picolinate, Vit C, Bet, and Tau has been reported to improve growth performance of broiler chickens exposed to heat stress in many previous experiments, however, none of these functional nutrients at the supplemental levels used in this experiment had a beneficial effect on broiler performance. This result was unexpected because dietary functional nutrients and their current supplemental levels were chosen based on previous researches reporting the positive effects of these functional nutrients on broiler performance under heat stress conditions. Thus, it is difficult to identify the clear reason; however, it may be attributed to the variations in animals and environmental conditions among the experiments because the extent of heat stress is influenced by both animal (e.g., sex, age, and genetics) and environmental factors (e.g., stocking density, ambient temperature and humidity, duration and the extent of heat stress, and rearing facility) [5,13]. However, we found that all functional nutrients used in this experiment showed the reduction in stress responses measured based on feather CORT concentrations and blood H:L. Therefore, it may be suggested that dietary supplementation of 0.5% Gln, 500 ppb Cr picolinate, 250 mg/kg Vit C, 0.2% Bet, and 1.0% Tau is effective in reducing stress responses of broiler chickens under heat stress conditions; however, these anti-heat stress effects are unlikely to lead to a full recovery of decreased growth performance of broiler chickens raised under the current heat stress conditions [45].

Heat stress has been reported to decrease meat quality of broiler chickens due to increasing oxidative stress and corticosteroid production [12,46,47]. In addition, it was reported that heat stress facilitated muscle glycogen breakdowns, which decreases meat pH, and therefore, lowering meat quality [47, 48]. A similar reduction in breast meat pH at 24 postmortem was observed in broiler chickens raised under heat stress conditions in this experiment. However, dietary functional nutrients had some positive effects on preventing meat pH reduction in this experiment. In general, the amounts of glycogen in the muscle and its degradation rate with conversion of glucose to lactic acid are the major determinants for the postmortem pH in the muscle [49]. Therefore, it appears that dietary functional nutrients used in this experiment may affect the amounts of glycogen and its breakdown rate to lactic acid in the muscle of broiler chickens although the clear mechanisms remain unknown. However, other meat quality measurements including WHC, meat color, and TBARS values were not affected by heat stress or dietary functional nutrients. In fact, the values for most of meat quality measurements in the current experiment fell within the normal quantitative range of broiler breast meat, indicating that the current heat stress conditions and dietary functional nutrients may have little effects on breast meat quality of broiler chickens.

Heat stress is well-known to depress immune systems because increased corticosteroid production and impaired immune organ development as caused by heat stress exerts a strong negative impact on immune systems [3,13,45]. Moreover, heat stress promotes oxidative stress, which increases lipid peroxidation of cell membrane of potential immune-related cells, leading to an impairment in immune responses [13]. In the present study, we also observed decreased CBH responses as a measure of cell-mediated immune responses in broiler chickens raised under heat stress conditions. Dietary functional nutrients including Gln [39], Cr [12,41], Vit C [13,14], Bet [16,43], and Tau [18] used in this experiment have been considered immunostimulants, especially for poultry exposed to heat stress. In the current experiment, however, feeding diets supplemented with 250 mg/kg Vit C showed a significant improvement in immune responses, which was similar to broiler chickens raised under thermoneutral conditions (i.e., PC group), whereas other functional nutrients did not show such a positive effect. The reason for these inconsistent results is not clear; however, it may be related to the variations in animals and environmental conditions among experiments, as seen in the results for growth performance in the present study. Moreover, different physiological mechanisms underlying the action of each functional nutrient whereby they modify immune responses may be one possible reason for the inconsistancy. For instance, Vit C can directly function as an antioxidant to protect against lipid peroxidation in immune-related cells [13,14], whereas other functional nutrients may have indirect actions on immune cell proliferation and activity by decreased corticosteroid production, increased immune organ development, and modulated nutrient metabolism [12,16,18]. Furthermore, it is also likely that the endogenous synthesis of Vit C in broiler chickens may be impaired or insufficient to satisfy increased requirement of Vit C to potentiate immune responses of broiler chickens exposed to heat stress; this may be the reason why dietary supplementation of 250 mg/kg Vit C exerted the positive effects on immune responses of broiler chickens [50]. However, it is still uncertain why other functional nutrients did not show such a positive effect as observed by dietary supplementation of Vit C. Therefore, more research is required to compare immune modulations by various functional nutrients in broiler diets under heat stress conditions.

Blood H:L and CORT concentrations have been reported to be elevated due to various stressors, and therefore, both measurements are typically considered potential stress biomarkers to evaluate magnitude of stress responses in poultry [13,51]. However, blood CORT concentrations have been often criticized due to a large diurnal variation and short half-life; this may render the measurement of the blood CORT concentrations inappropriate for a long-term stress response [13,29,51]. Therefore, feather CORT concentrations were measured in the present study because accumulatory properties of CORT in the feather can reflect a long-term stress response [29,52]. Our results indicated that all functional nutrients at the current supplemental levels decreased both feather CORT concentrations and blood H:L, indicating the reduction in stress responses of broiler chickens raised under the current heat stress condition. Surprisingly, the effect of dietary supplementation of 0.2% Bet on feather CORT concentrations and the effect of dietary supplementation of 1.0% Tau on blood H:L did not differ significantly from PC group. However, it is unknown why different functional nutrients influenced 2 stress biomarkers to the different extent. Increased CORT production in poultry due to various stressors is reported to induce impaired nutrient utilization, increased oxidative stress, and decreased immune responses, which are the primary causes of decreased productive performance, meat quality, and health of poultry [3,47,51]. However, we found no considerable positive effects on growth performance, meat quality, and immune responses of broiler chickens by feeding diets supplemented with 0.5% Gln, 500 ppb Cr picolinate, 250 mg/kg Vit C, 0.2% Bet, or 1.0% Tau, although all these functional nutrients decreased stress responses based on feather CORT and blood H:L. The reason is unclear; however, it appears that decreased stress responses by dietary functional nutrients at the current supplemental levels may hardly overcome the suppression of productive performance of broiler chickens raised under the current heat stress conditions. More research is required to evaluate the relationship between the extent of stress responses, such as tissue CORT concentrations and the reduction in productive performance of broiler chickens exposed to heat stress.

## CONCLUSION

Heat stress decreases growth performance, facilitates meat pH reduction, impairs immune responses, and increases stress responses in broiler chickens. However, dietary functional nutrients including 0.5% Gln, 500 ppb Cr picolinate, 250 mg/kg Vit C, 0.2% Bet, and 1.0% Tau have no positive effects on growth performance of broiler chickens raised under heat stress conditions used in this study. However, functional nutrients may exert the preventative effects on pH reduction in breast meat despite little effects on other meat qualities. Dietary supplementation of 250 mg/kg Vit C improves immune responses of broiler chickens, but other functional nutrients have no such an improvement in immune responses. All functional nutrients at the supplemental levels used in this study decrease stress responses of broiler chickens to a relatively similar extent based on feather CORT concentrations and blood H:L. However, this stress-reducing effect is likely insufficient to ameliorate decreased productive performance of broiler chickens raised under the current heat stress conditions.

## Figures and Tables

**Table 1 t1-ab-21-0230:** Composition and nutrient concentration of the basal diet

Items	Basal diet
Ingredients (%)	
Corn	58.71
Soybean meal (46% crude protein)	24.47
Corn gluten meal (61% crude protein)	5.77
Tallow	5.56
Mono-dicalcium phosphate	1.48
Limestone	1.21
DL-Methionine (98%)	0.32
L-Lysine HCl (78%)	0.58
L-Threonine (98.5%)	0.10
Choline (50%)	0.10
NaHCO_3_	0.10
Salt	0.20
Vitamin premix^1)^	0.10
Mineral premix^2)^	0.10
Celite	1.20
Total	100.00
Calculated energy and nutrient (%)	
AME_n_ (kcal/kg)	3,200
Crude protein (%)	19.54
Digestible lysine (%)	1.04
Digestible arginine (%)	1.10
Digestible tryptophan (%)	0.18
Digestible methionine + cysteine (%)	0.80
Digestible methionine (%)	0.54
Digestible threonine (%)	0.69
Total calcium (%)	0.79
Available phosphorus (%)	0.40

1) Provided per kg of the complete diet: vitamin A, 12,000 IU (retinyl acetate); vitamin D_3_, 4,000 IU; vitamin E, 80 mg; vitamin K_3_, 4.0 mg (menadione dimethpyrimidinol); vitamin B_1_, 4.0 mg; vitamin B_2_, 10 mg; vitamin B_6_, 6.0 mg; vitamin B_12_, 20.0 μg; calpan 20 mg; folic acid, 2.0 mg; biotin, 200 μg; niacin, 60 mg; antioxidant 2.0 mg; toyouserin 10.0 mg.

2) Provided per kg of the complete diet: manganese, 120 mg (MnO); copper 16.0 mg (CuSO_4_); zinc 100 mg (ZnSO_4_); iron, 60 mg (FeSO_4_); iodine, 1.25 mg (Ca[IO_3_]_2_); cobalt, 1.0 mg; selenium, 300 μg.

**Table 2:** Effects of dietary supplementation of various functional nutrients on growth performance of broiler chickens raised under heat stress conditions^1)^

Environment^2)^	Diet^3)^	Growth performance			

		BW (kg)	BWG (kg)	FI (kg)	FCR (kg/kg)
TN	Basal (PC)	2.75^a^	1.89^a^	3.23^a^	1.71
HS	Basal (NC)	2.39^bc^	1.52^bc^	2.62^b^	1.72
	Gln	2.42^b^	1.55^b^	2.70^b^	1.74
	Cr picolinate	2.33^c^	1.46^c^	2.63^b^	1.80
	Vit C	2.39^bc^	1.52^bc^	2.69^b^	1.77
	Bet	2.39^bc^	1.53^bc^	2.63^b^	1.72
	Tau	2.39^bc^	1.52^bc^	2.66^b^	1.75
SEM		0.268	0.272	0.438	0.025
p-value		<0.01	<0.01	<0.01	0.17

BW, body weight; BWG, body weight gain; FI, feed intake; FCR, feed conversion ratio; SEM, standard error of the means.

1) Data are least squares means of 6 observations per treatment.

2) TN, thermoneutral condition; HS, heat stress condition.

3) PC (positive control), basal diet; NC (negative control), basal diet; Gln, basal diet + 0.5% glutamine; Cr picolinate, basal diet + 500 ppb chromium picolinate; Vit C, basal diet + 250 mg/kg vitamin C; Bet, basal diet + 0.2% betaine; Tau, basal diet + 1.0% taurine.

a–c Means in the same column with different superscripts are different (p<0.05).

**Table 3:** Effects of dietary supplementation of various functional nutrients on breast meat quality of broiler chickens raised under heat stress conditions^1)^

Environment^2)^	Diet^3)^	Breast meat quality					

		pH, 24 h	WHC (%)	L*	a*	b*	TBARS
TN	Basal (PC)	6.17^a^	70.58	46.73	3.23	15.90	0.124
HS	Basal (NC)	5.55^c^	76.76	44.97	2.27	14.62	0.135
	Gln	5.93^ab^	70.85	44.90	2.62	14.20	0.121
	Cr picolinate	5.90^ab^	69.08	43.98	3.07	14.08	0.138
	Vit C	5.72^bc^	70.58	45.68	2.57	13.95	0.118
	Bet	5.95^ab^	68.29	45.63	3.98	16.63	0.137
	Tau	6.07^a^	68.36	45.85	3.28	15.68	0.134
SEM		0.104	2.211	1.257	0.496	0.820	0.011
p-value		<0.01	0.14	0.82	0.27	0.16	0.78

WHC, water holding capacity; L*, lightness; a*, redness; b*, yellowness; TBARS, thiobarbituric acid reactive substances; SEM, standard error of the means.

1) Data are least squares means of 6 observations per treatment.

2) TN, thermoneutral condition; HS, heat stress condition.

3) PC (positive control), basal diet; NC (negative control), basal diet; Gln, basal diet + 0.5% glutamine; Cr picolinate, basal diet + 500 ppb chromium picolinate; Vit C, basal diet + 250 mg/kg vitamin C; Bet, basal diet + 0.2% betaine; Tau, basal diet + 1.0% taurine.

a–c Means in the same column with different superscripts are different (p<0.05).

**Table 4:** Effects of dietary supplementation of various functional nutrients on cutaneous basophil hypersensitivity responses of broiler chickens raised under heat stress conditions^1)^

Environment^2)^	Diet^3)^	CBH responses		

		0 h	6 h	12 h
TN	Basal (PC)	0.00	0.73^ab^	0.80^a^
HS	Basal (NC)	0.00	0.65^b^	0.58^b^
	Gln	0.00	0.61^b^	0.53^b^
	Cr picolinate	0.00	0.35^c^	0.37^b^
	Vit C	0.00	0.93^a^	0.80^a^
	Bet	0.00	0.58^bc^	0.52^b^
	Tau	0.00	0.49^bc^	0.44^b^
SEM		-	0.095	0.077
p-value		-	<0.01	<0.01

CBH, cutaneous basophil hypersensitivity; SEM, standard error of the means.

1) Data are least squares means of 6 observations per treatment.

2) TN, thermoneutral condition; HS, heat stress condition.

3) PC (positive control), basal diet; NC (negative control), basal diet; Gln, basal diet + 0.5% glutamine; Cr picolinate, basal diet + 500 ppb chromium picolinate; Vit C, basal diet + 250 mg/kg vitamin C; Bet, basal diet + 0.2% betaine; Tau, basal diet + 1.0% taurine.

a–c Means with different superscripts within a column differ (p<0.05).

**Table 5:** Effects of dietary supplementation of various functional nutrients on stress biomarkers of broiler chickens raised under heat stress conditions^1)^

Environment^3)^	Diet^4)^	Stress biomarkers^2)^	

		Feather CORT (pg/mm)	Blood H:L
TN	Basal (PC)	7.38^c^	0.38^c^
HS	Basal (NC)	16.65^a^	0.75^a^
	Gln	11.82^b^	0.57^b^
	Cr picolinate	12.07^b^	0.58^b^
	Vit C	12.59^b^	0.61^ab^
	Bet	8.67^bc^	0.55^b^
	Tau	12.76^ab^	0.46^bc^
SEM		1.38	0.055
p-value		<0.01	<0.01

SEM, standard error of the means.

1) Data are least squares means of 6 observations per treatment.

2) Feather CORT, feather corticosterone (pg/mm feather length); Blood H:L, blood heterophil to lymphocyte ratio.

3) TN, thermoneutral condition; HS, heat stress condition.

4) PC (positive control), basal diet; NC (negative control), basal diet; Gln, basal diet + 0.5% glutamine; Cr picolinate, basal diet + 500 ppb chromium picolinate; Vit C, basal diet + 250 mg/kg vitamin C; Bet, basal diet + 0.2% betaine; Tau, basal diet + 1.0% taurine.

a–c Means with different superscripts within a column differ (p<0.05).
